# Conical diffraction illumination opens the way for low phototoxicity super-resolution imaging

**DOI:** 10.4161/cam.29358

**Published:** 2014-10-31

**Authors:** Julien Caron, Clément Fallet, Jean-Yves Tinevez, Lionel Moisan, L Philippe (Ori) Braitbart, Gabriel Y Sirat, Spencer L Shorte

**Affiliations:** 1 Bioaxial SAS; Paris, France; 2 PFID; Imagopole; Institut Pasteur; Paris, France; 3 Université Paris Descartes; Laboratoire MAP5; Paris, France

## Abstract

We present a new technology for super-resolution fluorescence imaging, based on conical diffraction. Conical diffraction is a linear, singular phenomenon, taking place when a laser beam is diffracted through a biaxial crystal. We use conical diffraction in a thin biaxial crystal to generate illumination patterns that are more compact than the classical Gaussian beam, and use them to generate a super-resolution imaging modality.

While there already exist several super-resolution modalities, our technology (biaxial super-resolution: BSR) is distinguished by the unique combination of several performance features. Using BSR super-resolution data are achieved using low light illumination significantly less than required for classical confocal imaging, which makes BSR ideal for live-cell, long-term time-lapse super-resolution imaging. Furthermore, no specific sample preparation is required, and any fluorophore can be used. Perhaps most exciting, improved resolution BSR-imaging resolution enhancement can be achieved with any type of objective no matter the magnification, numerical aperture, working distance, or the absence or presence of immersion medium.

In this article, we present the first implementation of BSR modality on a commercial confocal microscope. We acquire and analyze validation data, showing high quality super-resolved images of biological objects, and demonstrate the wide applicability of the technology. We report live-cell super-resolution imaging over a long period, and show that the light dose required for super-resolution imaging is far below the threshold likely to generate phototoxicity.

## Introduction

Far-field fluorescence super-resolution (SR) has matured over the past decade at a tremendous rate with a plethora of cutting-edge studies demonstrating the indisputable value of super-resolution imaging microscopy to studies in cell biology.^1^ This has in turn bred demand for routine utility, bringing today's state-of-the-art breadboard implementations to the first generation of commercial SR-imaging microscopy systems, making such techniques more conveniently available for wider use.^2^

Albeit powerful, techniques for SR microscopy have a toll charge: breaking the diffraction barrier is achieved through methods that impose constraints on the biological sample, and the experimental paradigm. Structured-illumination microscopy (SIM) techniques require capturing dozens of images to build a single super-resolution frame.^3,4^ STED-like techniques use a secondary high-power laser line to restrict fluorescence emission to a sub-diffraction area of the sample.^5^ Point-localization microscopy^6-8^ and photobleaching microscopy with nonlinear processing^9^ require repeated cycles of fluorophore activation, acquisition or bleaching to reconstruct SR representations of the sub-diffraction fluorophore distribution. It is therefore most notable for the case where living samples may be targeted by these SR microscopy techniques, that it will be the illumination regime that has perhaps the most significant consequences, including an increased propensity for phototoxic processes. This leads to generation of free radical species that certainly strain cellular metabolism, and in turn compromise complex cell functions such as sample motility/migration, signal-transduction, ECM adhesion dynamics, and ultimately even cell viability. Compounding the problem, SR-image acquisition has to occur with sufficient temporal resolution so as to be independent of cellular movements. Any physiological event must be sampled according to the Nyquist criterion, thus increasing further the risk of phototoxicity.^10^ Phototoxicity must be taken into consideration even in the case where slower illumination cycles apply, for example using SIM techniques the requirement for multiple frames increases the risk of cumulative phototoxicity^2^; whereas peak-power of STED lasers is the main risk factor for scanning devices known to induce toxic photochemical reactions.^11,12^

Despite the limitations outlined above, there are many groups reporting marked successes at super-resolution imaging^10^^,^ ^12-20^ in every case using highly specialized custom designed equipment, often confined to a very narrow spectrum of bespoke application. In many instances, only a limited number of frames could be acquired before the sample was too bleached or damaged to continue experimental imaging. Thus, to improve upon these previous works we present the bases to a novel super-resolution technique aimed to be of unique utility for long-term SR-imaging of live samples. The current implementation trades-off crude resolution performance for user-friendliness, applicability and more importantly, low phototoxic impact. It ships as a simple add-on for a commercial laser-scanning confocal microscope. It does not put constraints on the sample preparation and has a robust optic design. The principle used here allows considerably lower illumination light dose and peak power, even compared with standard fluorescence imaging, making it well suited for live cell SR-imaging over long time periods. Presenting here for the first-time the underlying principle of this entirely new SR-imaging method, we quantify its performance and discuss its applicability, especially its reduced phototoxicity impact that will open the way for SR-imaging of cell-migration in live cell experimental paradigms.

## Results

### System design

The super-resolution modality presented here is based on conical diffraction.^21-23^ Conical diffraction is an optical effect occurring when a beam propagates along the axis defined by the singularity of a biaxial crystal. For a single laser beam propagating in a thin crystal,^24^ it competes with classical diffraction, creating a beam shaping effect. The exact nature of the resulting light distribution is determined by the polarization states at the entry and the exit of the crystal. We propose a system that exploits this effect to achieve super-resolution imaging.

**Figure 1:**
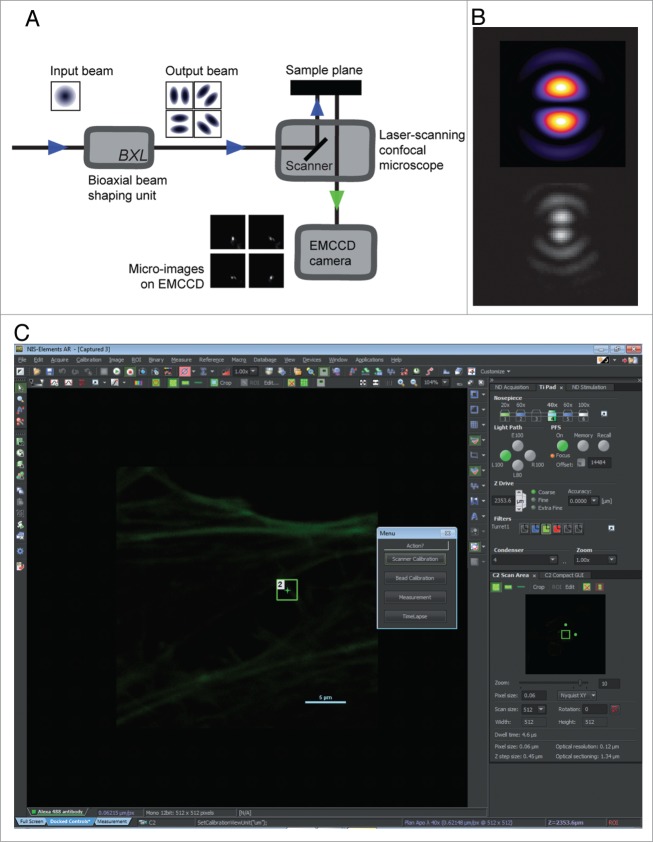
**(See previous page).** A super-resolution system based on conical diffraction. (**A**) Overview of the system. The laser fiber input of a classical commercial laser-scanning microscope is hijacked to pass through the BSM. This module shapes a Gaussian beam in a “half-moon” shape, characterized by two compact lobes. Four orientations of the lobes are used throughout this paper. Each point of the sample is then illuminated by the four half-moon patterns in quick succession. A non-descanned EMCCD camera, generating one micro-image per illumination per point, captures the resulting fluorescence image. (**B**) Simulation (top) and measurement (bottom) of one orientation of the illumination pattern used for super-resolution. The measure is done by capturing a slightly defocused reflection on the coverslip, which slightly alter the pattern shape by stressing the outer rings. (**C**) The super-resolution module transparently integrates in the existing software that controls the normal operation of the laser scanning confocal microscope. Super-resolution imaging is operated thanks to a LabView program, triggered by macros implemented directly in the NIS-Element software. The user is asked to draw a rectangular ROI on the sample image, which will then be scanned in super-resolution mode.

The implementation described here consists of a single Beam-Shaping Module (BSM) that plugs on a commercial laser-scanning confocal microscope (LSCM, Nikon C2, Nikon Corporation, Tokyo, Japan). An optical fiber is diverted from the LSCM laser bench, and plugged into the beam-shaping module containing all the supplemental optics (**Fig. 1a**). It consists of a thin biaxial crystal, a polarization state generator (PSG) and a polarization state analyzer (PSA). The PSG is made of a linear polarizer followed by a pair of Pockels cells (LEYSOP, Bristol, UK); the PSA is laid out identically in reverse order. All the Pockels cells are controlled electronically, allowing the control of polarization without any moving mechanical parts in the unit. The two polarization states are chosen so that the biaxial crystal generates a specific pattern (**Fig. 1b**), which differs from the fundamental Gaussian beam. It is made of two lobes, more compact than the classical PSF, with a marked dark stripe separating them. These lobes display high spatial frequencies and a good contrast, and have therefore the desired properties to be used in super-resolution. The resulting beam is then coupled back to the scanner of the confocal microscope that is used to scan the sample, point by point. Each point of the sample is sequentially illuminated using four orientations of the pattern. The resulting emission is captured on a fast, high sensitivity camera (ImagEM C-9100–13, Hamamatsu Photonics, Hamamatsu, Japan), mounted to the rear port of the microscope through a 4x relay lens. The acquisition of a region made of WxH scan points of the sample therefore generates WxHx4 micro-images. To retrieve the super-resolution information from these micro-images, a dedicated algorithm processes them. Prior to acquiring data, the 4 illumination patterns are calibrated once by scanning a single fluorescent bead (ø 100 nm, TetraSpeck, Life technologies). The micro-images and the illumination patterns are then fed to the reconstruction algorithm, which makes use of the structured illumination reconstruction then exploits the local nature of excitation and the known calibrated topology of the patterns to localize further fluorophore emitters. For the images presented in this paper, the reconstruction typically took 30 min.

Both classical imaging and super-resolution imaging are performed through the native software (NIS-Elements, Nikon, **Figure 1c**). Macro instructions in the NIS-Elements software, coupled to a custom software module (LabView, National Instruments, Austin, Texas, USA) were used to control the different optic elements (laser, cameras, polarization control). In a typical use-case, the user acquires a standard image of the sample through classical imaging, draws a region-of-interest (ROI) on the image, and captures a super-resolution view of this region.

### Validation, fidelity and performance of the super-resolution imaging modality

**Figure 2.**
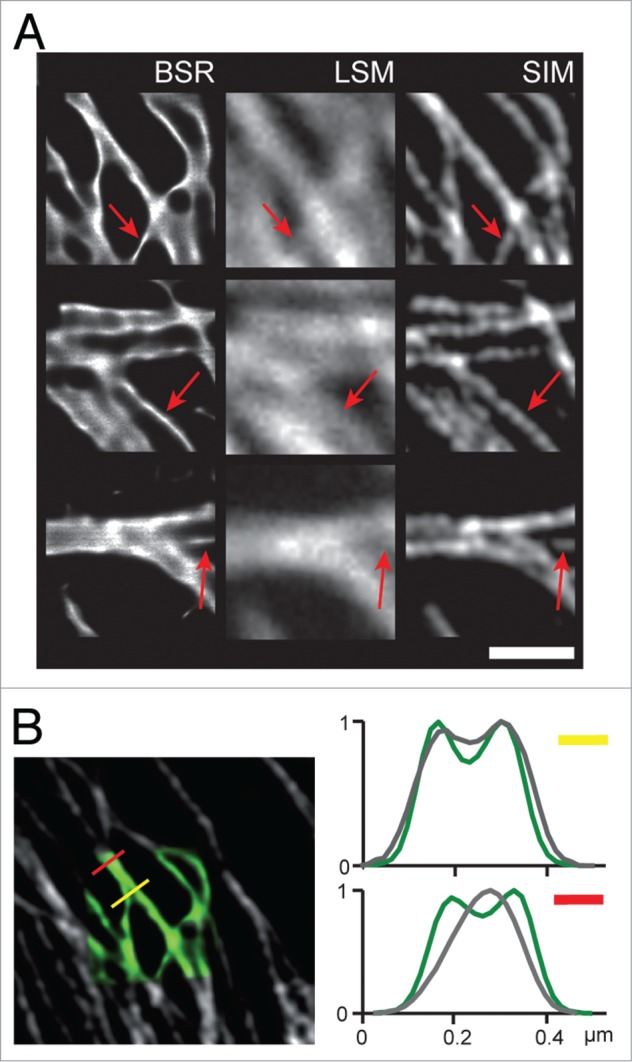
Super-resolution performance of the BSR modality. Glioma cells (U373) were stained with antiTubulin-Alexa488, and imaged using a 60x water NA = 1.2 objective. (**A**) Three 2 μm x 2 μm ROIs of a glioma cell imaged using from left to right: the BSR modality (BSR); the laser scanning confocal used in classical mode (LSM); a 3D-SIM microscope with a 63x oil objective NA = 1.4 (SIM). Scale bar: 1 μm. Red arrows point to key image features. (**B**) Left: Overlay of the first patch imaged using the BSR mode (green) and the 3D-SIM mode (gray). Right: intensity profiles corresponding to the yellow and red lines on the left image, taken across the junction of two microtubules.

We first imaged fixed Glioma cells (U373) stained with antiTubulin-Alexa488, using a 60x water NA = 1.2 objective. Super-resolution images (**Fig. 2a**, BSR, left column), then LSCM images (central column) of the same regions were acquired. The super-resolution images were almost devoid of noise thanks to the denoising step inherent to the reconstruction process and microtubules appeared as sharp, thin highly contrasted structures. Classical LSC images have the features of direct, classical imaging: The microtubules appear as noisy, broad structures of ∼300 nm, compatible with the lateral diffraction limit of 250 nm for confocal images excited by a 488 nm laser beam. The BSR images highlight numerous structures that are hidden in the LSC images (**Fig. 2a**, compare red arrows).

To confirm the reconstructed structures were not corrupted by spurious artifacts, we imaged the same areas with a 3D-SIM microscope (ELYRA, Carl Zeiss AG, Jena, Germany) using a 63x oil NA = 1.4 objective (**Fig. 2a**, SIM, right column). Images obtained with the two super-resolution modalities tested (BSR and 3D-SIM) were consistent. Every structure found in the BSR image corresponded to comparable structure in the SIM image. The BSR images however appear sharper and less noisy. The minor discrepancies in the thickness of structures observed between the BSR and SIM modality are likely to originate from the 3D geometry of the sample: The SIM images are optically sectioned and super-resolved in 3D, whereas here the BSR modality is limited to a single 2D plane acquisition. As soon as a microtubule moves slightly out of the focal plane, its BSR image will be soiled by out-of-focus blur. Nonetheless, the comparison with the three imaging modalities (BSR, SIM and LSC) demonstrates that the BSR modality does not introduce artifactual structures.

To further quantify the BSR modality fidelity, we investigated whether the intensities in the reconstructed BSR images are faithful to the actual fluorophore distribution in the sample. We used the SIM images as ground truth, carefully aligned each BSR ROI with the corresponding SIM region, and calculated ρ, the Pearson linear correlation coefficient between the intensities in the SIM image vs. the BSR image for all pixels of a ROI (see Materials and Methods and **Figure S1**). We found ρ to be 0.69, 0.56 and 0.87 for the 3 ROIs, values significantly different from 0. It is important to note that this is a pessimistic estimate for linear correlation: the values are negatively affected by the noise in the SIM image, as well as out-of-focus blur and difference in super-resolution performance. Moreover, possible registration errors induce dramatic adverse effects on the linear correlation. The large values we find for ρ demonstrate that the BSR intensity depends linearly in the fluorophore concentration in the sample.

We assessed the super-resolution performance of the BSR modality using the Rayleigh criterion. We carefully aligned the SIM image and the BSR image of the same region (**Fig. 2b**, left). Two crossing microtubules were then followed from their junction until they could be separated with at least a 26% dip between the two peaks in an intensity profile across the microtubules section (**Fig. 2b**, right, yellow profile). On the BSR image, the two peaks were separated by 130 nm, well below the classical resolution limit for the objective used. At the same position, the profile obtained with the SIM image displays a dip of only 15%, showing that the Rayleigh criterion is not met for this modality. Another profile on the other side of the junction (**Fig. 2b**, red profile) revealed the BSR modality able to resolve two microtubules separated by 130 nm with a 21% dip in intensity, whereas the SIM reported just one structure profile, demonstrating superior performance of conical-diffraction based super-resolution.

### Applicability of conical diffraction-based super-resolution

**Figure 3.**
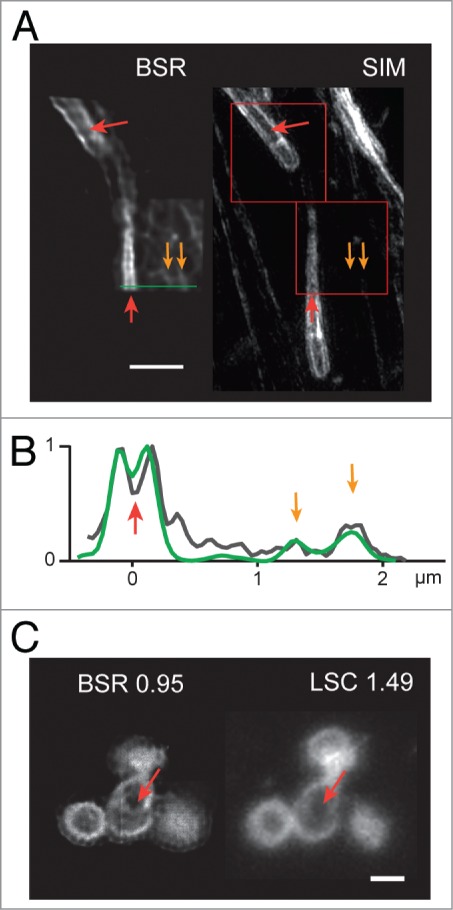
(**A**) Super-resolution images of the bacterium *R.conorii* infecting HeLa cells, stained using Phalloidin-Alexa488. Left: two 3 μm x 3μm patches of a cell, imaged using the BSR mode with a 40x air objective NA = 0.95. Right: The same area imaged using a 3D SIM microscope, with a 63x oil objective NA = 1.4. Scale bar: 2 μm. Red and orange arrows point to key features, see text. (**B**) Intensity profile along the green line for the BSR modality (green) and the 3D SIM modality (gray). Red and orange arrows match to the image features in panel **A**. (**C**) Left: BSR imaging of a foci of *Neisseria meningitidis*, using the 40x air objective NA = 0.95. Right: Classical laser-scanning confocal imaging of the same foci, using a 100x oil objective NA = 1.49. Scale bar: 1 μm. Red arrows point to a thin membrane that separates two bacteria in the central clump.

The beam-shaping module relies on a biaxial crystal to generate the patterns that foster super-resolution. As such, the super-resolution capabilities of the system do not depend on a specific microscope configuration, and can be achieved with any kind of microscope objective. **Figure 3** demonstrates this flexibility. Fixed samples were acquired for super-resolution using an air objective (40x air, NA = 0.95). Though normally not accessible with this objective using classical imaging, the super-resolution images render the hollow structure of the actin comets of *R.conorii* (**Fig. 3a**, red arrows). The 3D-SIM microscopy images show that the BSR technique can emulate the results obtained through an objective with a superior numerical aperture. These images also demonstrate the sensitivity and the wide dynamic range of the technique: The intensity profile of the BSR image clearly highlights the actin bundles of the HeLa cell hosting the bacterial infection, though their intensity range is considerably smaller (**Fig. 3a** and **b**, orange arrows). On the 3D-SIM intensity images and profile, the same actin bundles are difficult to visualize, their signal being somewhat drowned by noise, their presence is more difficult to ascertain compared with BSR imaging even with the lower performance objective.

**Figure 3c** compares the BSR modality based on a relatively low NA air objective, with classical LSCM detection using a relatively higher-performance oil objective based upon its higher numerical aperture. The latter setup is considered optimal for studies on small biological objects close to the classical resolution limit, such as bacterial clumps. Again the BSR modality using low numerical aperture air objectives nonetheless performs optimally making the classical resolution limit target scale fully accessible. Pertinently, once the required spatial resolution is achieved through either means, an extra effort must be made to generate data of sufficient quality, by taming the image noise inherent to both modalities. Because we compare here the BSR modality based on a sensitive camera vs. the LSCM modality that is based upon a classical PMT setup, we see on this pair of images that the BSR modality outperforms LSCM by allowing to make the right conclusion *i.e.* the central structure of this clump imaged on the BSR modality revealing two bacteria separated by a faint, thin and otherwise hard to resolve membrane, whereas with the LSCM modality suffering both noise and diminished sensitivity entirely misses these structures (**Fig. 3c**, red arrows).

### Non-invasive long-term live cell super-resolution imaging

**Figure 4.**
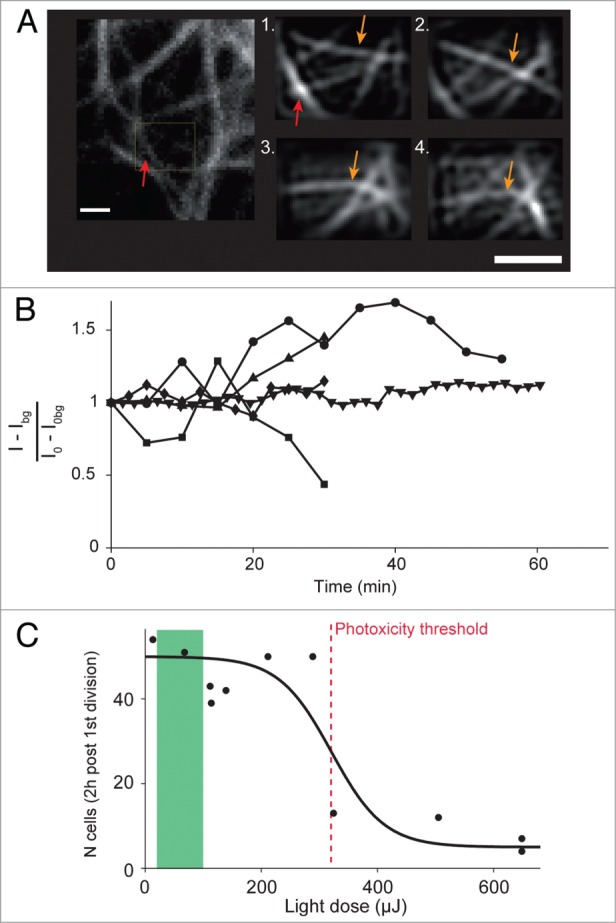
Live cell super-resolution imaging and photodamage induced by BSR imaging. (**A**) Example of time-lapse imaging of live cells stained for microtubules. Left insert: view of the region of interest, imaged with the classical LSC modality prior to time-lapse imaging. The yellow rectangle depicts the ROI for super-resolution acquisition. Numbered inserts: images of the ROI acquired with the BSR modality over time. 1: t = 0 min. 2: t = 2:30 min. 3: t = 9 min. 4: t = 18 min. Red arrows point to a microtubule in both the LSC and BSR image. Orange arrows follow the same microtubule over time. (**B**) Live cell super-resolution time-lapse imaging and photobleaching. The curves report the mean raw intensity above background, normalized with respect to t = 0, for the super-resolution ROI over time. Cells stained for microtubules using tub-eGFP or EMTB-3G were used indifferently. Curves variations reflect cytoskeleton dynamics rather than photobleaching. (**C**) Phototoxicity curve for a laser scanning confocal, see Material and Methods. Red dashed line: phototoxicity threshold for a LSCM. The green area depicts the range of light doses used for BSR imaging throughout this paper.

The BSR modality requires only a few μW of light intensity at the sample level to operate, much less than required by some other super-resolution techniques.^1^ This small amount of light nonetheless allows it to generate images of good quality, which makes it well suited to study live specimen in super-resolution. To support this assertion, we imaged U373 live cells stained for microtubules thanks to a transfection with tubulin-eGFP or EMTB-3G.^25^ We implemented in the LabView software module a scanning mode dedicated to the imaging of a region in quick succession (see Materials and Methods). Super-resolution images were reconstructed post-acquisition. In some cases, we acquired a large field of view of the cell between each SR acquisition, using the classical confocal mode. An example of such an experiment is depicted in **Figure 4a**. The confocal and SR images are in perfect agreement, and demonstrate that BSR is suitable to render super-resolution on a live specimen. Both modalities show that the cells remain motile and active during the whole acquisition (movie S1 and movie S2). The microtubules are dynamic, which indicates that the cell migration is not visibly perturbed by the acquisition. A plot of the mean fluorescence intensity measured in the SR region over time displays variation that reflects the cell dynamics, rather than the typical decay associated with fluorophore bleaching, which also indicates that the BSR illumination exerted very little photobleaching (**Fig. 4b**).

However, emission intensity measurements are only indirect reporters of photodamage. The relevant variable to monitor for photodamage is the light dose, which is the amount of light sent on the sample measured in units of energy during imaging. Assessing the phototoxic impact of an illumination modality in a quantitative way is a difficult task. We recently reported a method^26^ consisting in imaging the development of a *C.elegans* embryo using the inspected illumination modality. From the first anaphase at a constant temperature of 21 °C, the normal development of the embryo produces 50 cells after 2 h. Imaging this process with a high light dose slows down or arrests the normal development, which results in having a lower number of cells at the end of the two hours period. Here, the photodamage is quantified through counting the number of cells for a given light dose, after two hours of imaging. Because the BSR modality is based on a LSCM, we generated the phototoxicity curve for a laser-scanning based illumination modality, using classical imaging with a 488 nm continuous laser, as described in ref. 26 (**Fig. 4c**). We find the phototoxic threshold to be at 320 μJ every 2 min for a pixel dwell time of 1.58 μs/pixel. The BSR images generated in this section were acquired with a power measured at the sample level of 1–2 μW. With a total exposure of 2.4 s, we shine 2.4–4.8 μJ on the sample for every acquisition. The live experiments were performed with a temporal rate of at most 1 image every minute. In the least favorable case, we yield 10 μJ every 2 min at most, well below the phototoxic threshold.

### Discussion

Conical diffraction-based super-resolution is a novel imaging technique that exploits a phenomenon seldom used in light microscopy. It generates super-resolved images of the sample, whose intensities follow linearly the fluorophore concentration. This makes the BSR technique directly applicable to all analysis and quantification methodologies based on intensities. It also brings several unique features that make it an attractive technology.

### A flexible super-resolution platform

#### Retrofitting existing installation

The system presented here achieves super-resolution thanks to a beam-shaping module in the form of a relatively small footprint, tabletop box containing all the required optical elements that sits next to the confocal microscope. This design makes it comparatively facile to retrofit any existing LSCM installation, and thereby opens the way for super-resolution capabilities to most any classical point-scanning confocal imaging system. In the current study, the beam-shaping module exit was opto-mechanically coupled to the scanner entry through a coupling lens, which had to be manually aligned. The next iteration of the system will include laser fiber input and output. As such, retrofitting will be feasible on all LSCM that couple their laser source to the scanner unit with a single optical fiber, provided that the scanner optics doesn't alter the beam polarization; the BSM will simply plug between the two. In such a configuration, the capabilities and performance of the standard confocal mode can be fully preserved inasmuch as the BSM can be tuned in a manner leaving the input beam profile unmodified. Evidently, a further “dealbreaker” requirement for a LSCM configuration to be BSM-BSR compatible is to have a free port on the microscope to which the BSR detection camera can be mounted.

Power losses in the BSM are inherent to the beam shaping effect used in the module. These losses set a limit on the minimal power that must be delivered by the laser source of the target equipment. The last element of the BSM polarization state analyzer being a polarizer, losses about a factor of two; and further marginal losses are expected with the seven optical elements (lenses, Pockels cells) that comprise the BSM, which must also be taken into account. The transmittance of these elements was measured to be around 90%, therefore an extra loss factor of two has to be considered. In total, we can estimate that the BSM transmits about 25% of the light power from the laser sources. However, these losses have very little consequences in practice, as the power required by our technique at the sample level is in the range of only a few μW, compared with a few hundreds of μW typically used in classical LSCM imaging. Consequently, the laser sources currently available on commercial platforms all exceed by far the range of power required.

#### Unconstrained super-resolution

The BSM can work nominally with probes excited in the range 405–640 nm and with an emission in the range 420–800 nm, the latter being limited only by the objective transmittance and the camera sensitivity. Super-Resolution is achieved through projecting local structured patterns onto the sample, which qualifies it as a super-resolution structured-illumination technique. Because imaging is based on plain linear fluorescence, the BSR modality does not require a special sample preparation, a staining protocol or specific dyes to operate. It performs with any probe that can be used with classical confocal imaging. Also, it does not set extra constraints on the illumination light path: the only requirement for the optical setup is to transduce faithfully the patterns. The super-resolution modality is therefore usable with any objective, for any magnification, low or high numerical aperture and also for any immersion medium: oil, water or air. The same goes for multi-channel acquisition: the number of different channels that can be sequentially acquired on the system is set only by the available excitation lasers, dichroics and filters in the light path.

Beyond the self-evident reduced cost advantage there are more specific application advantages using the BSR modality employing low numerical aperture air objectives, as opposed to optimized high numerical aperture immersion objectives and classical imaging. Notably, cryogenic correlative-light-electron-microscopy (Cryo-CLEM) is limited to the use of air objectives when customized cryo-stages are used on the light microscope^27^ because of the need to maintain the objective physically separated by a long working distance from the cryogenic chamber. However, by virtue of its ability to optimize the use of air objectives to produce high resolution imaging BSR promises to liberate Cryo-CLEM imaging from its current limitations in classical optical set-ups combing cryo-stage imaging on light microscopes. This utility is further appreciated when one considers that BSR imaging using air objectives also facilitates automated microscopy, wherein an air objective is amenable to scanning adjacent areas of a sample without the constraint to replace immersion medium that must otherwise be topped-up during region-of-interest displacement.

### A light-efficient super-resolution modality with a low phototoxic impact

The detection light path of the BSR system is simple: it consists of a dichroic, an emission filter, a relay lens and a camera. The specific camera we picked is a very sensitive EM-CCD with a nominal quantum-efficiency (QE) above 90%. Compared with the 20–30% QE of the PMT on which the confocal imaging depends, one can expect a much improved sensitivity, particularly when imaging faintly labeled structures. The main disadvantage brought by this detector is its readout time. To properly reconstruct a super-resolution image, we need to capture the micro-image generated by illuminating the sample with each pattern. Even with a fast camera, it is unlikely that we can go well below 1 ms of exposure for each pixel, compared with the pixel dwell-time of the order of a few μs achieved when using PMTs. This makes this super-resolution modality slow compared with PMT-based techniques. To work around this limitation, we typically use small ROIs, only generating super-resolution images across a restricted field-of-view (FOV) as done with some other modalities.^12,19,20^ The larger, contextually important, field of view is generated on our system using the confocal mode. This apparent drawback - rather long pixel dwell-time, compensated through small FOV - brings interesting side-benefits. First, with a longer pixel-dwell time, the illumination power must comparatively be smaller for the same light dose. Our system therefore works with very low peak powers. As stated above, this facilitates retrofitting: power losses in the BSM are modest, which considerably lowers power requirements on laser output. More importantly, a low illumination power is crucial for non-invasive imaging. Indeed, the fluorescent light-induced damage critically depends on the peak power delivered to the sample, even for an equivalent light dose.^11,26,28^ Second, because we scan the sample point by point, the light dose is proportional to the area scanned. By using a small ROIs, we illuminate only the parts of the sample that are important for super-resolution imaging, hereby reducing the light dose, economizing the light-budget and in turn protecting the living biological sample from potential photodamage. These frugal needs in peak-power and light-dose should not be underestimated and certainly make the BSR modality especially suitable for non-invasive, long-term super-resolution imaging of living samples.

The light dose requirement derived here allows comparing the BSR technique with classical imaging modalities. Calculations drawn from ref. 26, using the same methodology as in this work, indicate that the phototoxic light dose for standard wide-field imaging is around 15 to 50 mJ/cm^2^ per acquisition. This is in good agreement with the values found afterwards by other investigators with different specimens.^29^ Retaining the value of 20 mJ/cm^2^ and using an order of magnitude for the typical area of a cell of 500 μm^2^, we get a light dose estimate of about 0.1 μJ to acquire a single image of a cell with a wide-field microscope. This must be compared with the phototoxic light dose for LSCM measured here of 320 μJ for a full stack. The acquisition of a 2 × 1.6 μm^2^ region in BSR involves in the least favorable case about 10 μJ. However, ordering performance using these numbers is not fully relevant: The LSCM dose is calculated for the acquisition of a full optically sectioned Z-stack image; the wide-field dose is calculated for a single plane; the BSR dose is calculated for a 2D patch of 2 × 1.6 μm^2^. Nevertheless we see that the BSR technique photodamage impact is commensurate with classical imaging modalities rather than with other SR imaging modalities.

Interestingly, this light dose is used very efficiently. First, the detector is a very efficient light detecting camera, ensuring an exquisite sensitivity and enabling unmatched image quality, even in weakly labeled areas of the sample (see for example **Figure 4a**, compare left and right panels). Second, BSR relies on a linear fluorescence effect. In BSR mode, the fluorophores are illuminated by such a low light power that they work in a linear regime, and the photon number at detector level is directly proportional to the illuminating light dose. This is unlike STED and PALM/STORM techniques, where a considerable portion of the energy shone on the sample is used to build the SR image, but does not contribute directly to final image brightness. For the BSR modality like for the SIM technique, each photon arriving at the sample contributes to the final SR image quality.

### Conical-diffraction-based microscopy in motion: 3D live cell imaging

The present paper restricts itself to 2D super-resolution imaging. Several developments, all based on the phenomenon of conical-diffraction in thin biaxial crystals are on the way.

First, the super-resolution imaging can be extended to 3D imaging. The images are optically sectioned and can benefit from a specialized 3D reconstruction algorithm that would combine the information from micro-images acquired at different Z positions. This will readily generate 3D images super-resolved in 2D, and optically sectioned. Moving to true 3D super-resolution will require exploiting new beam shapes, that vary rapidly in Z, opening the way to the third-dimension using the same super-resolution principle described here in 2D. Interestingly, this can happen without any change of the hardware, as the biaxial crystal and the PSGs can already generate all the required patterns.

Second, it will be interesting to test how this super-resolution modality behaves in thick and dense samples. Other SR techniques are known to quickly deteriorate as the ROI moves further away from the coverslip inside a thick sample. As the BSR modality is based on LSCM, one expects it to reproduce the penetration depth of classical confocal imaging, and image in SR mode beyond 40 μm deep into the sample. Further, a two-photon version of BSR could double or even quadruple this penetration performance while maintaining SR quality.

## Materials and methods

### Preparation of samples for imaging

For live cell imaging (**Fig. 4a** **and** **b**, **movie S1** and **movie S2**), Glioma cells (U373) were transfected by electroporation of 2 μg DNA, encoding either for tubulin-eGFP or EMTB-3G,^25^ 48 h before imaging. Cells were then plated in glass bottom petri dishes containing a 50–50% mix of DMEM and F12 media, with 10% FBS, penicillin/streptomycin and nonessential amino acids.

For fixed sample imaging, Glioma cells (U373) were treated 5 min with methanol at -20°, and labeled with rat anti-tubulin (AbD Serotec). *Rickettsia conorii* actin comets were prepared as described in ref. 30. *Neisseria meningitidis* samples were prepared as described in ref. 31.

### Acquisition parameters

The system was configured to acquire a SR view of patches of size ranging from 2 × 1.6 μm^2^ to 7 × 7 μm^2^. Depending on the objective chosen, the spacing between each point scanned varied, and determined the number of micro-images required for the reconstruction. For the data presented in this work, there were: 441 scanning points therefore 1764 micro-images for the live tubulin movies; bacteria images used 3 ROIs, each of 900 scanning points, amounting to a total of 10800 micro-images; fixed tubulin images used 900 scanning points therefore 3600 micro-images; whereas each ROI of the actin comet images used 400 scanning points and 1600 micro-images.

### Image fidelity assessment

Linearity of intensities between the BSR and SIM modalities was assessed by first carefully aligning images of the same region of the same sample using each modality. Registration was done automatically by linear alignment using the SIFT plugin^32^ of Fiji software.^33^ Pearson linear correlation between pixel intensities of the two aligned images was then calculated using MATLAB (The MathWorks, Natick, USA).

### Live cell imaging

A specific hardware control system was built to achieve a superior frame rate for live cell experiments. Instead of scanning the region point-by-point the scanner was used in line-scan continuous mode, but pulsing the laser at discrete times. We rely on an auxiliary fast monochrome camera (EPIX SV9M001M, Epix Inc., Buffalo Grove, IL, USA) mounted on the second backport of the Nikon Ti Eclipse microscope to achieve a good precision in the illumination spot localization. A custom made cube in the second filter wheel allowed to divert 4% of the emitted light onto this secondary camera that was used to record the laser spot location over time. The control software synchronized laser pulses, camera acquisition and Pockels cells during a normal scan of the C2 confocal scanner, and record synchronized pairs of images, for both the EMCCD camera and the auxiliary camera. The position of the laser spot was measured with a precision of typically 20 nm. In this scanning mode, the four orientations of the pattern are not positioned at the same sample location, but these locations are recorded with high precision by the auxiliary camera. The algorithm was redesigned to adapt to this condition with minimal loss of performances, allowing 2 × 1.6 μm^2^ region to be imaged every 20 s within which the laser was triggered on for a total duration of 2.4 s, so that each of the 1600 micro-images was illuminated for 1.5 ms.

**Figure 4a** and movie S1 and movie S2, cells were filmed for 30 min, acquiring alternately first one 80×80 μm^2^ region using the classical LSCM modality and then one 2 × 1.6 μm^2^ region using the BSR modality. The sequences were recorded with the 40x air objective (NA = 0.95).

### Phototoxicity curve and phototoxicity threshold

**Figure 4c** displays a phototoxicity curve generated following ref. 26. Each black dot represents the result of imaging a *C.elegans* embryo over two hours from the onset of its first cell division, using one 3D stack every 2 min. The X-axis reports the light dose per stack used during imaging. The Y-axis reports the number of cells in the embryo after two hours of imaging. The incident light power was measured by placing the probe of a laser power meter (FieldMaxII-TO, Coherent Inc., Santa Clara, CA, USA) at the focal plane of the objective. The light dose is simply derived by multiplying this power by the total time the laser is on during a full acquisition. At 21 °C, a non-illuminated embryo develops into 50 cells. The phototoxicity threshold is defined as the light dose above which the development is impaired enough so that the embryo only has half of the expected number of cells. The threshold is measured by fitting a sigmoidal curve to the points, and was found in this case to be 320 μJ.

## Supplementary Material

2013CAM0088R1-Sup.pdf
