# Supplementary material for: Conical diffraction illumination opens the way for low phototoxicity super-resolution imaging
Source: Cell Adh Migr. 2014 Oct 31;8(5):430–9. doi: 10.4161/cam.29358 (PMC4594584; doi:10.4161/cam.29358)
Supplement: 2013CAM0088R1-Sup.pdf [file kcam-08-05-969636-s001.pdf]

Landes Bioscience
www.landesbioscience.com

Supplemental Material to:

Julien Caron, Clément Fallet, Jean-Yves Tinevez, Lionel Moisan, L Philipp e Braitbart, Gabriel Y Sirat, Spencer L Shorte

Conical diffraction illumination opens the way for low phototoxicity super-resolution imaging

Cell Adhesion & Migration 2013; 8(4)
http://dx.doi.org/10.4161/cam.29358

http://www.landesbioscience.com/journals/celladhesion/article/29358/

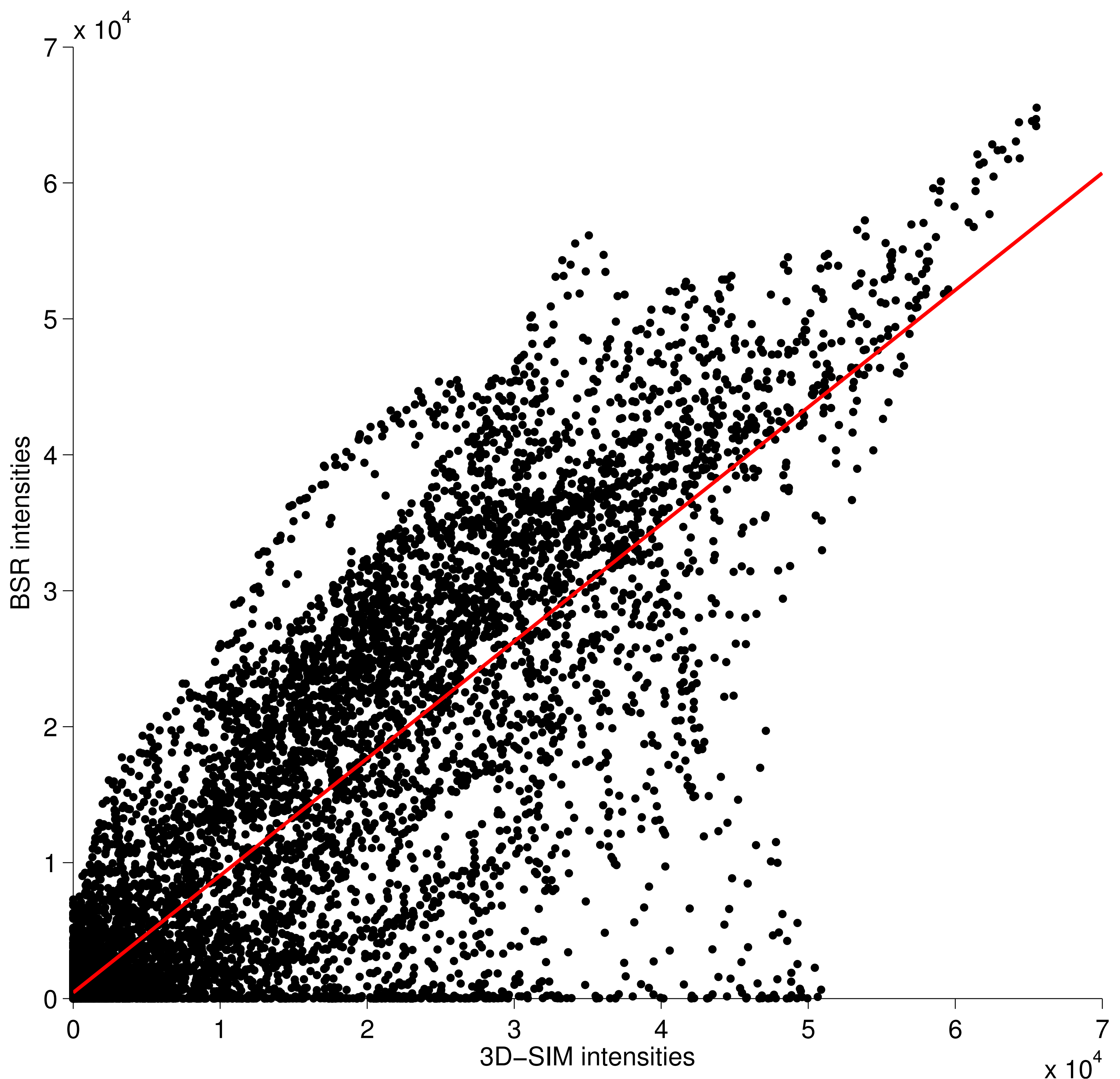

x 10^4
7
6
5
4
3
2
1
0
BSR intensities
0
1
2
3
4
5
6
7
3D−SIM intensities
x 10^4

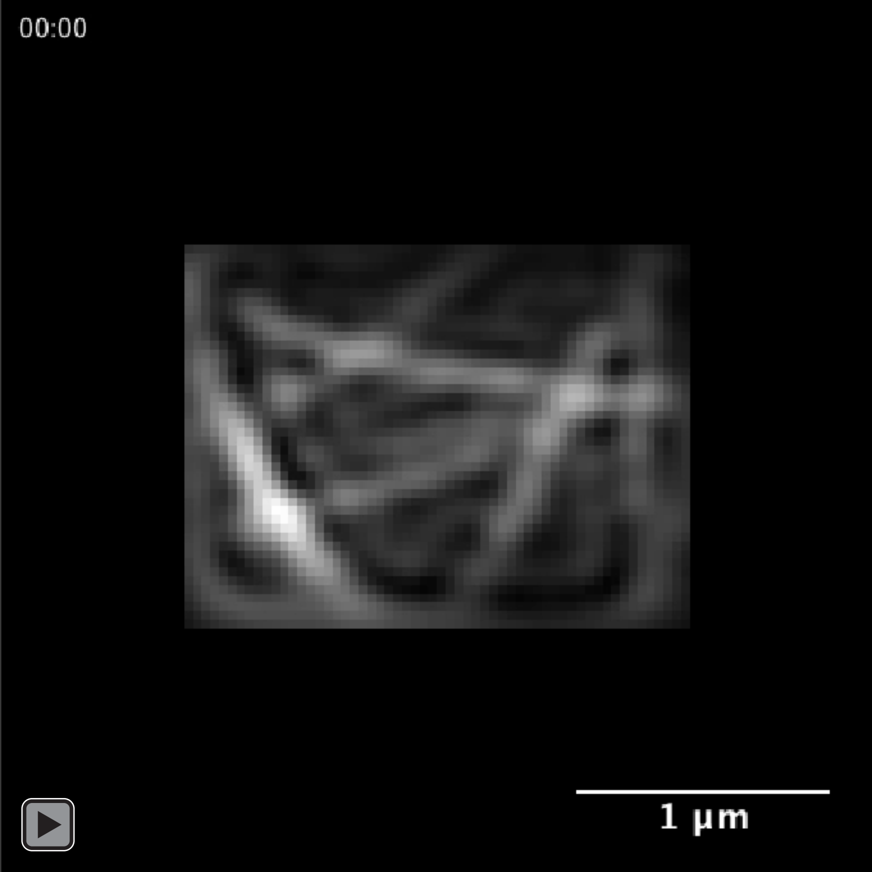
00:00
1 µm

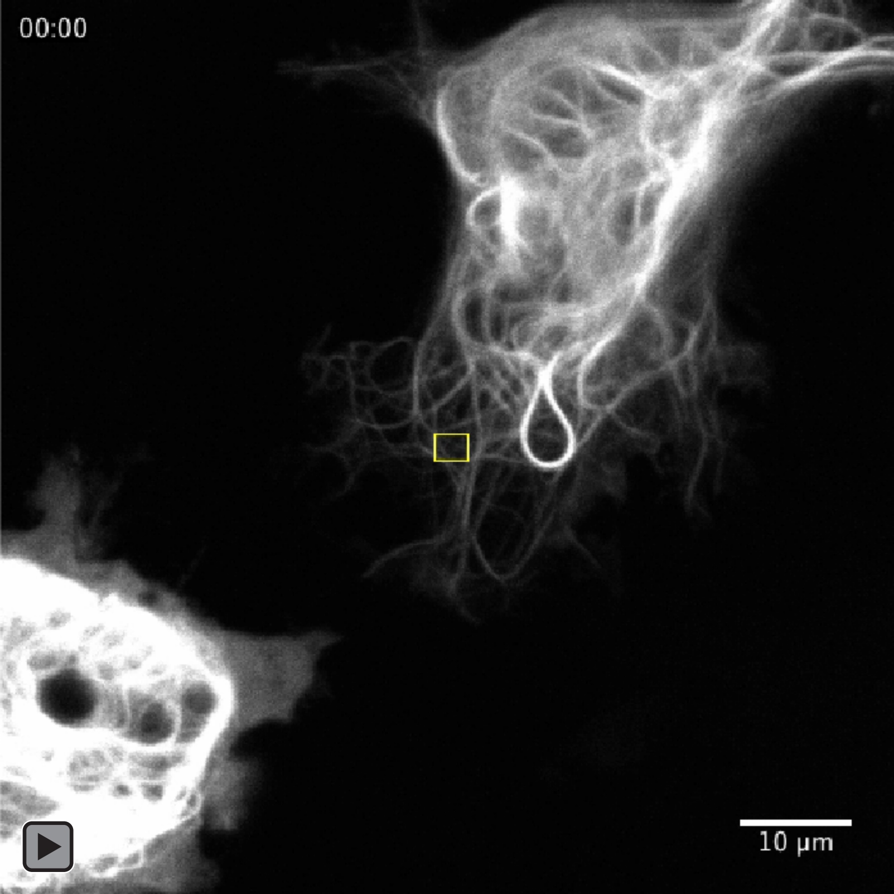
00:00
10 µm

Supplemental Figure 1.

Linearity of reconstructed pixel intensities. The BSR intensities from a ROI from Figure 2a were plotted against the intensities of the SIM image of the same region. The red line pictures a linear fit with a R^2 value of 0.76.

Supplemental Movie 1.

Live cell imaging using the LSC modality for the cell of Figure 4a. Each frame is separated by 2:30 minutes. After each acquisition, a frame was collected using the BSR modality to generate the Supplemental Movie 2.

Supplemental Movie 2.

Live cell imaging using the BSR modality for the cell of Figure 4a. Each frame is separated by 2:30 minutes, and was acquired after the matching frame of the Supplemental Movie 1.
